# Experimental approaches to investigate biophysical interactions between homeodomain transcription factors and DNA

**DOI:** 10.1016/j.bbagrm.2024.195074

**Published:** 2024-12-05

**Authors:** Fadwa Mekkaoui, Robert A. Drewell, Jacqueline M. Dresch, Donald E. Spratt

**Affiliations:** a Gustaf H. Carlson School of Chemistry and Biochemistry, Clark University, 950 Main Street, Worcester, MA 01610, United States of America; b Biology Department, Clark University, 950 Main Street, Worcester, MA 01610, United States of America

**Keywords:** Homeodomain, Transcription factor, *Drosophila*, Binding affinity, Biophysics, Protein-DNA

## Abstract

Homeodomain transcription factors (TFs) bind to specific DNA sequences to regulate the expression of target genes. Structural work has provided insight into molecular identities and aided in unraveling structural features of these TFs. However, the detailed affinity and specificity by which these TFs bind to DNA sequences is still largely unknown. Qualitative methods, such as DNA footprinting, Electrophoretic Mobility Shift Assays (EMSAs), Systematic Evolution of Ligands by Exponential Enrichment (SELEX), Bacterial One Hybrid (B1H) systems, Surface Plasmon Resonance (SPR), and Protein Binding Microarrays (PBMs) have been widely used to investigate the biochemical characteristics of TF-DNA binding events. In addition to these qualitative methods, bioinformatic approaches have also assisted in TF binding site discovery. Here we discuss the advantages and limitations of these different approaches, as well as the benefits of utilizing more quantitative approaches, such as Mechanically Induced Trapping of Molecular Interactions (MITOMI), Microscale Thermophoresis (MST) and Isothermal Titration Calorimetry (ITC), in determining the biophysical basis of binding specificity of TF-DNA complexes and improving upon existing computational approaches aimed at affinity predictions.

## 1. Background

### 1.1. Homeodomain transcription factors

Eukaryotic gene regulatory networks are controlled by transcription factors (TFs) that bind to specific DNA sequences within the genome. Once bound, TFs contribute to the complex transcriptional regulation of target genes [1]. Many studies have demonstrated that TFs often bind to a range of target DNA sequences with varying affinity [2,3], can form complexes with cofactor proteins [4], and can either activate or repress transcription of a target gene in a context-dependent manner [5,6]. While the importance of TFs in the genetic control of cellular function and embryonic development is well established [7], the biophysical and thermodynamic basis for where, when and how a TF binds to a specific DNA regulatory sequence remains poorly understood.

**Fig. 1.**
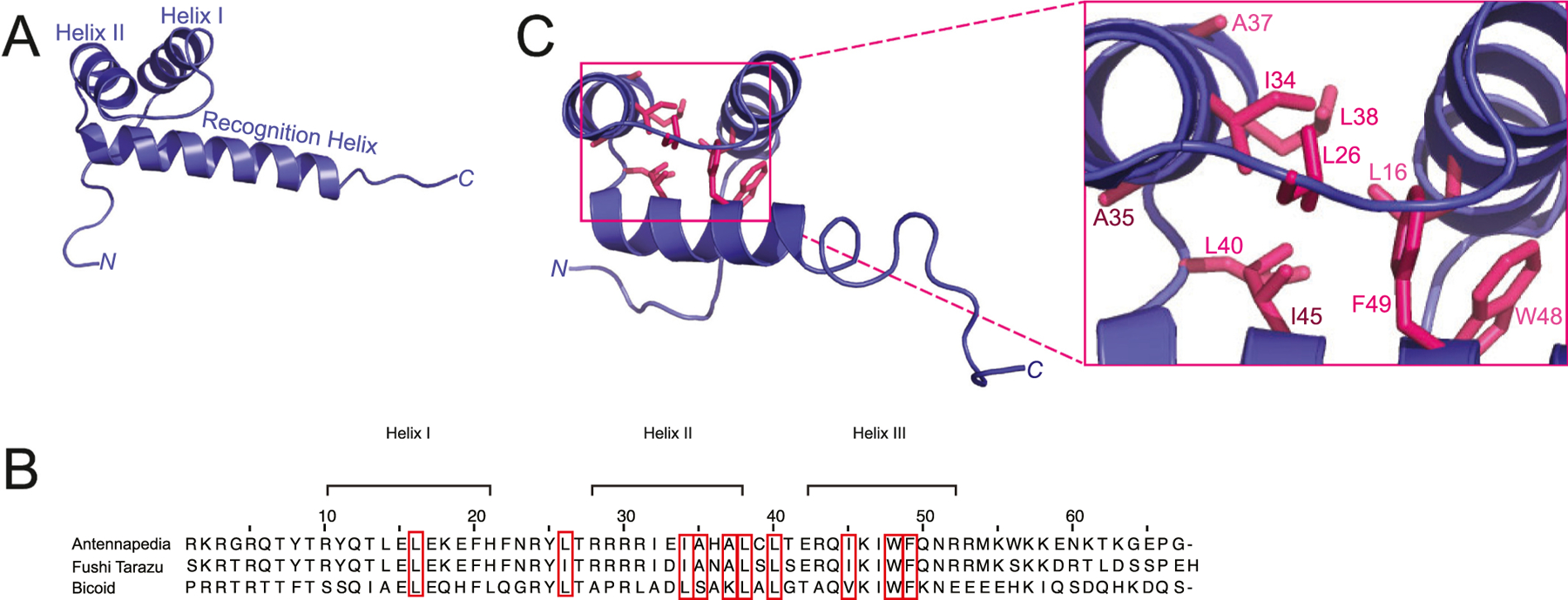
Transcription Factor Homeodomain (TF-HD) Structure. (A) 60 amino acid helix-turn-helix DNA binding domain [163,164]. The HD consists of three alpha helices – Helix I, II and II. Helix III is also referred to as the recognition helix, due to its role in direct binding to DNA [165]. (B) Comparison of TF-HDs BCD, ANTP, and FTZ. Multiple sequence alignment displaying the boundaries of the three α-helices. Residues in the hydrophobic core of the protein are designated with a red box [20,166]. (C) Conserved residues found in the hydrophobic core (shown in pink) and the HD are shown in blue. The HD of ANTP (PDB 1HOM) [25] was visualized using Pymol [167]. (For interpretation of the references to colour in this figure legend, the reader is referred to the web version of this article.)

The biophysical organization of TF proteins has long been recognized as modular [8], with each different protein consisting of a DNA recognition domain and additional structural and functional effector domains [9]. Researchers have classified TF proteins into distinct families based on the shared molecular characteristics of their DNA binding domain [10]. One such DNA binding domain is the homeodomain (HD), which is found in an estimated 15–30 % of all TFs in eukaryotes, depending on the species analyzed [11]. The HD is a 60 amino acid residue motif encoded by a 180 bp homeobox DNA sequence in the corresponding TF protein-coding gene. The homeobox was first discovered in 1984 in *Drosophila melanogaster* [11] and has since been identified in a number of eukaryotic organisms, including yeast, mouse and human [12,13]. Structural studies over the last four decades have revealed that the HD forms a highly conserved helix-turn-helix DNA binding domain consisting of three α-helices and an unstructured *N*-terminal extension (Fig. 1a) [11,14].

The homeobox gene family is large and plays important roles in diverse biological processes. For example, the HD TF proteins regulate cellular differentiation and function, and are critical during embryonic development in metazoans [11,12]. A class of homeobox genes, the HOM-C complex in *Drosophila*, and their mammalian homologs, the Hox genes, are organized in linked chromosomal clusters and exhibit similar expression patterns along the anterior-posterior axis, which determine the basic body plan during embryogenesis [15]. HD TFs regulate a wide range of developmental networks by controlling the temporal, spatial and cell type-specific expression of target genes, including the nervous system [16] and eye development [17]. Due to their important evolutionarily conserved roles in embryonic development, significant biophysical and structural work has been performed on HD proteins in an effort to expand our understanding of their diverse functions, characterize the molecular activity of these TFs, and improve predictions for TF-DNA binding events.

### 1.2. Structural characterization of HD TFs

**Fig. 2.**
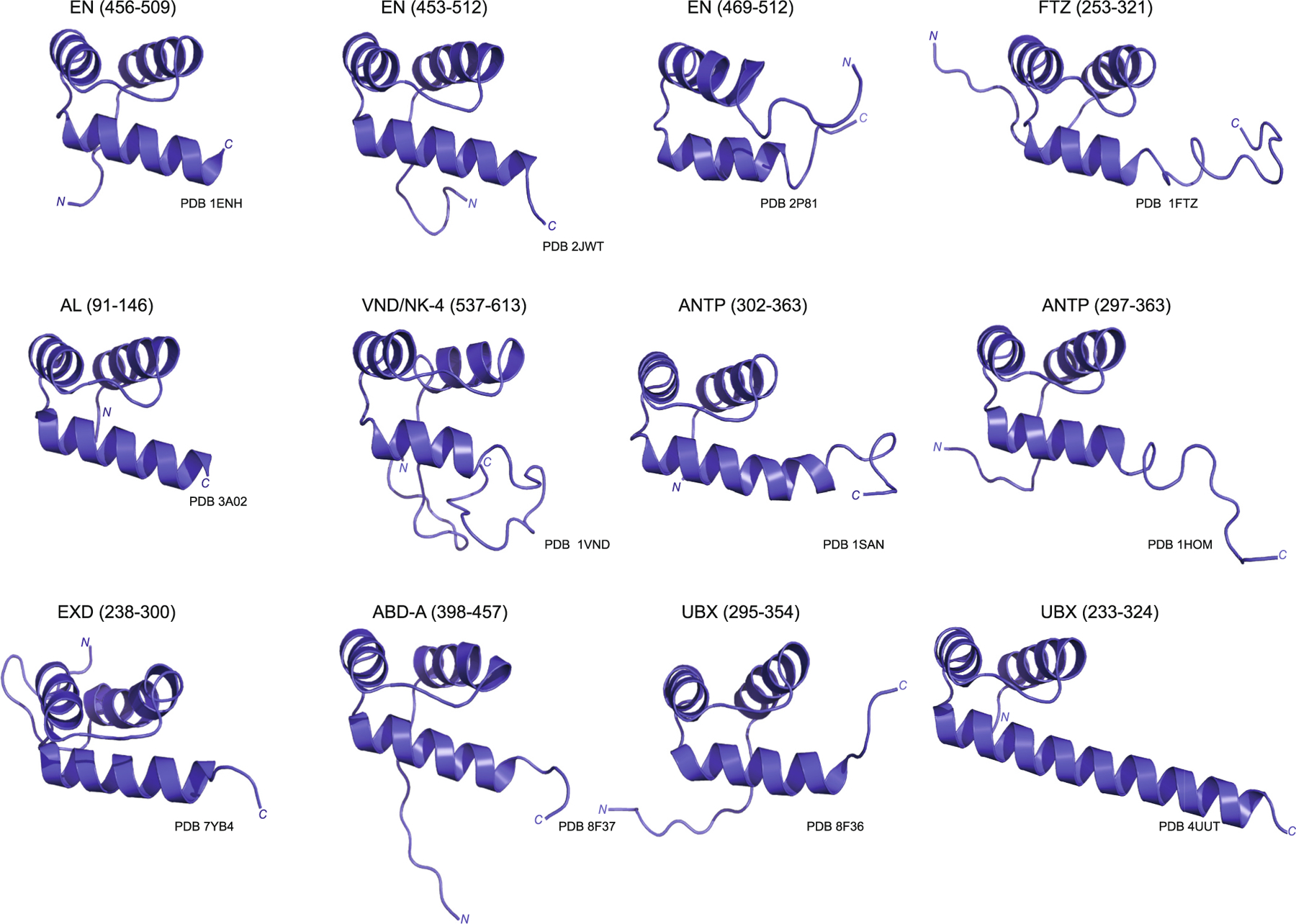
Solved Transcription Factor-Homeodomain (TF-HD) structures. Overlayed structures of various TF-HDs show the similarities and differences between the homeodomains. All of these structures show the three helices that are characteristic of the HD family, with variations being caused by in the number of extra residues included at the *N-* and *C*-termini, and length of the recognition helix.

In 1989, the first 3D structure of the HD of a TF was solved for *D. melanogaster* ANTENNAPEDIA (ANTP), a member of the HOM-C complex, using homonuclear Nuclear Magnetic Resonance (NMR) spectroscopy [18]. This ensemble of structures revealed three α-helices, with helices I and II sitting anti-parallel to one another and connected by a loop while helix III, commonly referred to as the *recognition helix*, was positioned perpendicular to the other two helices (Fig. 1a) [19]. These studies also uncovered the boundaries of the three α-helices (I – residues 10–21; II – residues 28–38; III – residues 42–52, see Fig. 1b), as well as the hydrophobic core holding the HD together (specifically in ANTP – L16, L26, I34, A35, A37, L38, L40, I45, W48, and F49, see Fig. 1c) [18]. In a subsequent study, Qian et al. determined a very similar structure for FUSHI TARAZU (FTZ) (Fig. 2), a pair-rule TF responsible for establishing the correct number of body segments during *Drosophila* embryogenesis with 85 % sequence identity to ANTP in the HD [20]. In 2006, the solution NMR structure of a third HD protein, BICOID (BCD) revealed a nearly identical organization to ANTP and FTZ, with some variance in the unstructured *N*-terminal arm of BCD (residues 2–10; RRTRTTFTS) that can be attributed to greater sequence variability in this region of the protein (Fig. 1b) [21]. These early structural studies provided valuable atomic level detail and indicated a clear rationale for how these HD TFs might bind to DNA to regulate target genes.

The shared structural organization of the HD observed in ANTP, FTZ and BCD has now been characterized in a number of other members of the HD TF family in *Drosophila* (Fig. 2). These studies reveal that all of the *Drosophila* HDs studied to date (listed in Table 1) consist of the three canonical α-helices (Figs. 1a and 2).

**Fig. 3.**
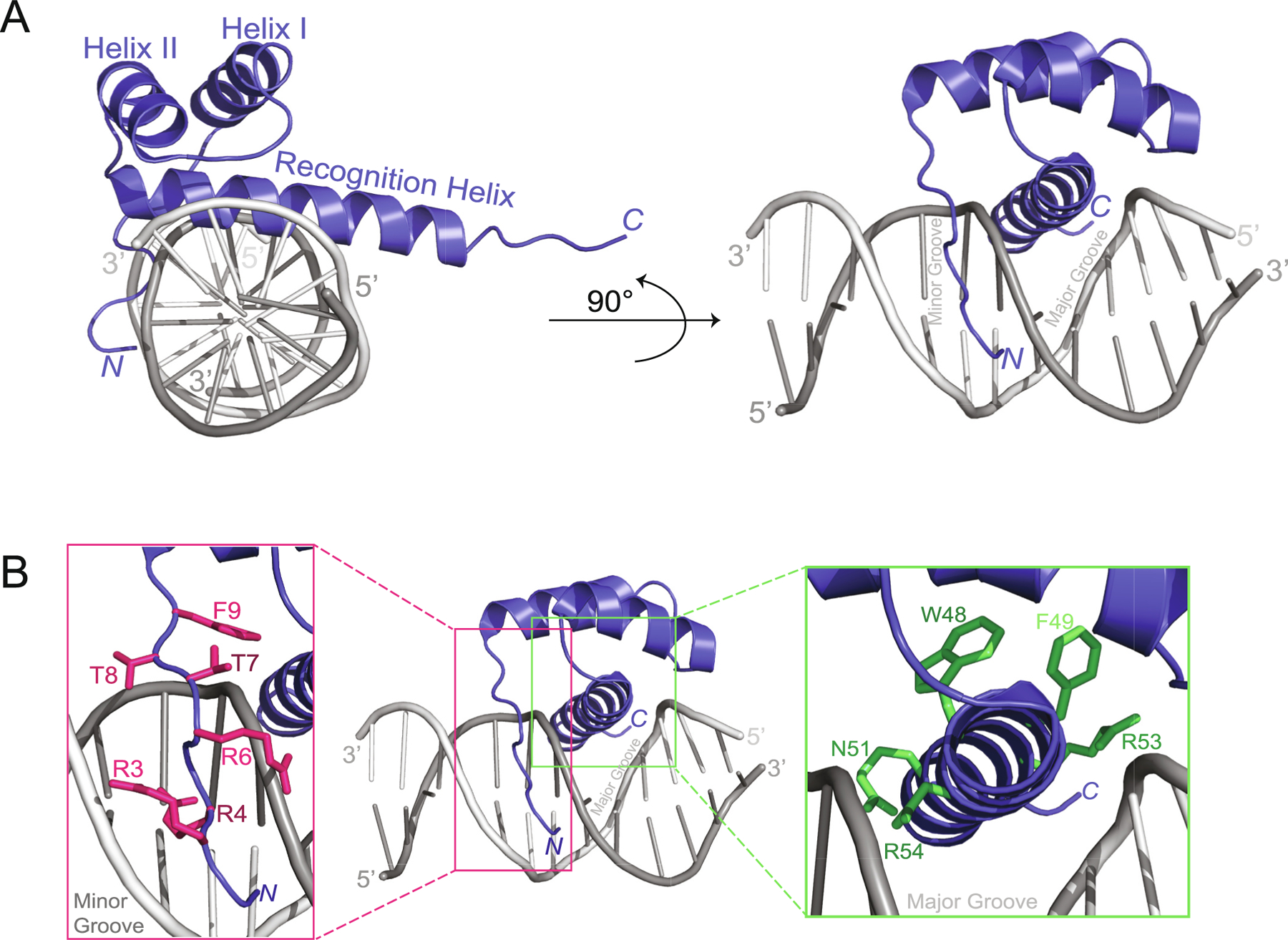
ANTP TF-HD bound to double stranded DNA. (A) Top view of double-stranded DNA shows Helix III (aka the “recognition helix”) bound to DNA. When rotated 90 degrees counter clockwise, it becomes apparent that the recognition helix inserts into the major groove of DNA. (B) Important residues in the TF-DNA complex. The structure shows that the *N*-terminal arm of the HD makes contact with the minor groove, while the recognition helix binds to the major groove of DNA [165]. Residues R3, R4, R6, T7, T8, F9 on the *N*-terminal arm (shown in pink) make contact with the DNA minor groove [164]. Residues W48, F49, N51, R53, and K/R54 (shown in green) are highly conserved in the TF-HD recognition helix and play a critical role in HD-DNA binding [45]. The ANTP HD-DNA complex (PDB 1ZQ3) [166] was visualized using Pymol [167]. (For interpretation of the references to colour in this figure legend, the reader is referred to the web version of this article.)

**Fig. 4.**
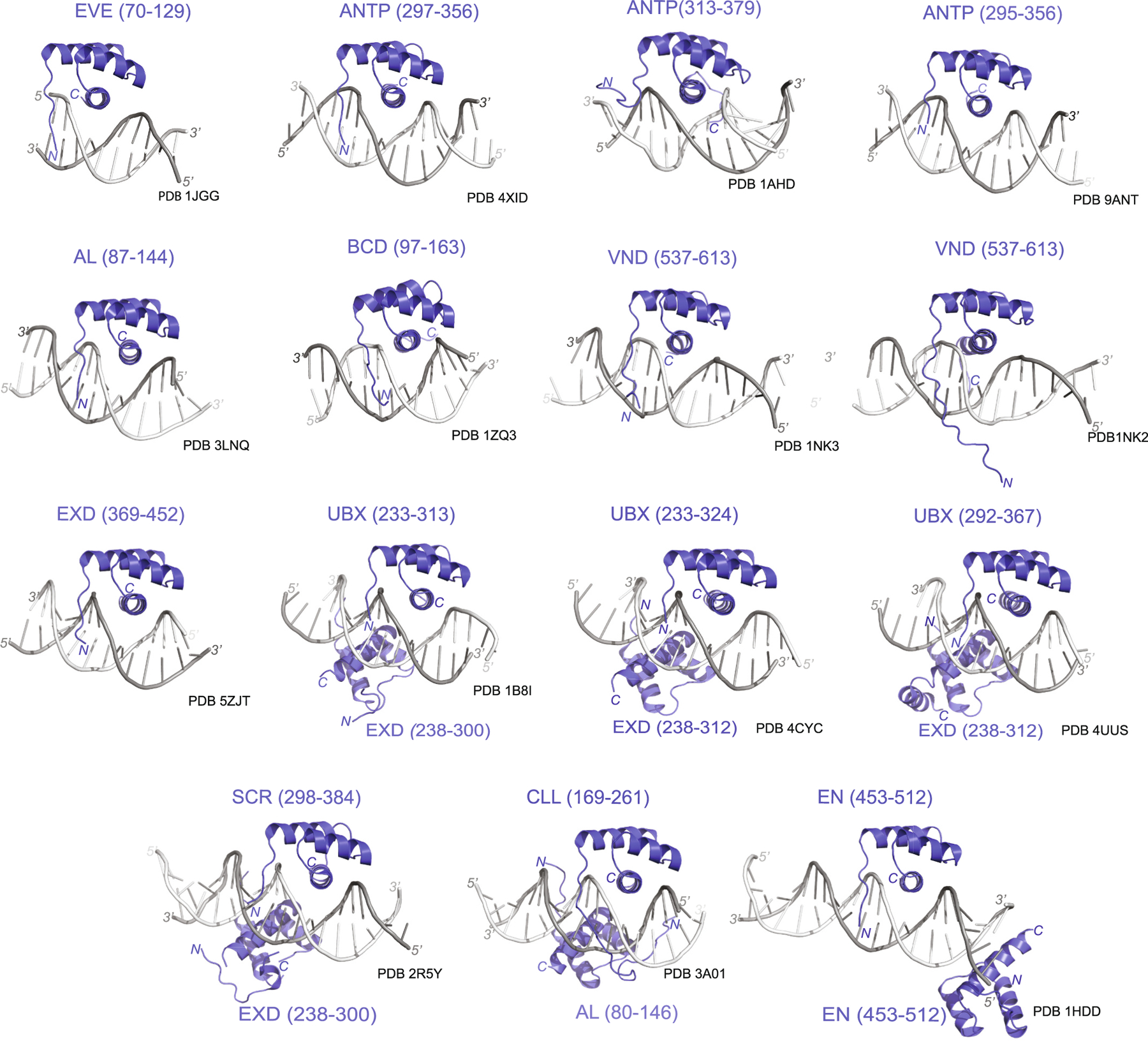
Solved Transcription Factor-Homeodomain DNA structures. Overlayed structures of various TF-HD (blue) bound to DNA (grey) show the similarities and differences between the homeodomains. The HD recognition helix can be seen binding to the major groove of DNA in each example, with some TFs requiring a cofactor (typically EXTRADENTICLE) to bind to the DNA sequence. (For interpretation of the references to colour in this figure legend, the reader is referred to the web version of this article.)

Furthermore, NMR spectroscopy and X-ray crystallography have been used to elucidate not only the global fold HD alone for many of these TFs (Fig. 2), but also TF in complex with DNA (Fig. 3) and TF-DNA complexes with other HD cofactors present (Fig. 4). These approaches have also revealed the similar 3D structures for other eukaryotes and prokaryotes homeodomains (summarized in Tables S1 and S2).

### 1.3. Previous structural studies of HDs bound to DNA

Structural studies have provided valuable information regarding HD-DNA interactions and the DNA binding specificity of HD TFs. ANTP was the first HD-DNA complex structurally examined using both NMR and X-ray crystallography [41]. Both methods demonstrated that ANTP achieves specificity through rapidly fluctuating DNA contacts and preferentially binds to the core consensus DNA sequence 5′-TAATGG-3′ [42]. As expected, both the X-ray and NMR structures showed that the recognition helix binds in the major groove of DNA. In these structures, I47 was observed to make important contacts with the methyl group of thymine 4 (TAATGG) and the C8 of adenine 3 (TAATGG) [42]. N51 also forms a pair of hydrogen bonds to adenine 3, while Q50 is involved in van der Waals interactions with cytosine 6 and hydrogen bonding with the phosphate backbone (Fig. 3b). Intriguingly, some of the structures in the NMR ensembles also suggest that the HD *N*-terminal extension engages in the adjacent minor groove (Fig. 3b). In these solution structures, R5 of the HD *N*-terminal extension forms a hydrogen bond to thymine 1 (TAATGG), fitting into the minor groove (Fig. 3b). These results suggest that HD TFs can potentially experience different conformations in solution, but both the X-ray and NMR structural data demonstrate consistent docking patterns within a HD-DNA complex. In the last two decades, the structure of several *Drosophila* HD TF-DNA complexes have been characterized in detail (Table 1). All of these structures demonstrate a strikingly similar organization with the recognition helix binding in the major groove of DNA and additional residue contacts from the HD *N*-terminal region in the adjacent minor groove (Fig. 4).

More recent studies have also shown that HD TFs can bind cooperatively to DNA to potentially modulate site specificities and functional output [43,44]. HD TFs also have the ability to regulate transcription by interacting with each other in homodimeric or heterodimeric configurations (Fig. 4) [43]. For example, BCD monomers interact cooperatively with each other to bind DNA and regulate spatial transcription of various target genes [43]. *Hunchback* (*hb*) is one such target gene that is activated in a threshold dependent response to the concentration gradient of the maternally deposited BCD protein in the early embryo. Activation of the *hb* gene requires the cooperative binding of BCD to multiple DNA binding sites within regulatory regions and the resulting spatial expression profile of *hb* helps to establish the anterio-posterior axis in the embryo [45].

In summary, structural studies confirmed that the recognition helix (helix III) binds within the major groove of DNA, while residues in the *N*-terminal arm, located prior to helix I of the HD, appear to wrap around DNA and make contact with the minor groove (Fig. 3) [21]. The reported binding affinities of these HD-DNA complexes are thought to be a function of the base pair sequences within the specific DNA binding site [46] and may also bind cooperatively via protein-protein interactions. Given the overall similarity of the structural organization of HD TF interactions with DNA and the clear conservation of amino acid residues within HDs, a central question for the field is how individual HD TFs exhibit DNA sequence binding specificity and consequently regulate the expression of different target genes. By improving our understanding of the biophysical basis for HD-DNA complex formation, we can further examine binding affinity, investigate how this affinity may be linked to the shape of DNA, improve upon computational predictions, and ultimately elucidate HD-DNA sequence specificity.

### 1.4. Integrating in silico approaches to characterize TF-DNA interactions

As high quality gene expression data has become more prevalent, the ability of researchers to build accurate mathematical models of the functional activity of TFs within regulatory networks has also increased at an unprecedented rate. We are not only seeing more precise model fits to existing molecular experimental data, but we are developing a wealth of approaches utilizing mathematical tools to unravel the complex regulatory mechanisms involved in gene regulation, including protein-DNA interactions, protein-protein cooperativity, short-range repression, and dual activator/repressor roles of TFs [47–59].

**Fig. 5.**
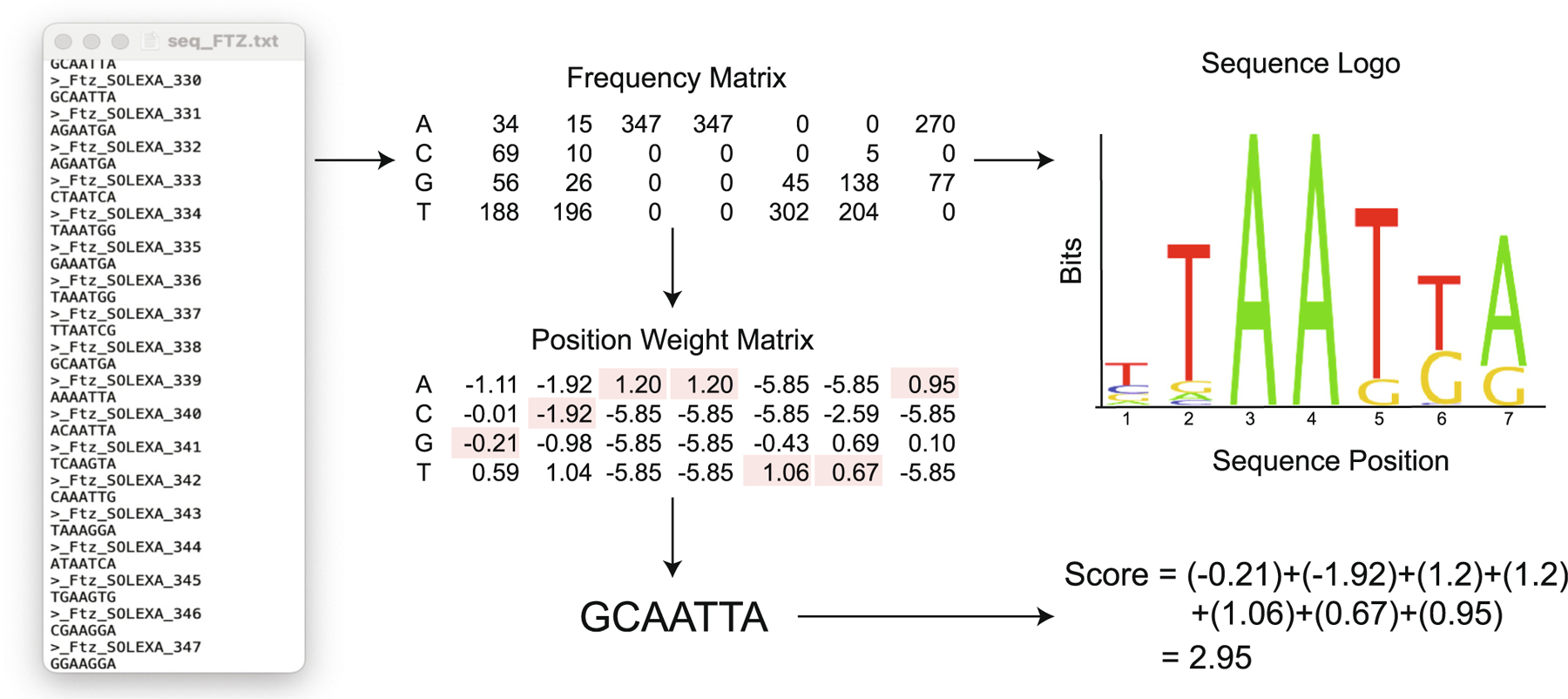
Position Weight Matrix (PWM)-based approaches. Simple schematic of PWM-based approaches (using FTZ protein as an example). All PWM-based approaches require a list of known binding sequences for the protein of interest, shown here in a single FASTA file (seq_FTZ.txt). From this set of sequences, a frequency matrix is constructed that contains the number of times each nucleotide is observed at each position within the binding site. The frequency matrix can then be used to create a graphical visualization of the individual nucleotide preferences at each position, referred to as a sequence logo. In addition, the frequency matrix is converted to a PWM using a logarithmic transformation. Note that this transformation often includes some adjustment for the background frequencies for each of the four nucleotides in the genome and the total number of known binding sequences. The PWM shown has been constructed using log-odds frequencies compared to background frequencies of A:0.3, C:0.2, G:0.2, and T:0.3. This PWM is then used to score individual sequences of interest. An example sequence (5′-GCAATTA-3′) is shown below the PWM along with the entries from the PWM used in calculating the sequence’s corresponding score, 2.95, highlighted in red. (For interpretation of the references to colour in this figure legend, the reader is referred to the web version of this article.)

For these mathematical models to accurately predict transcriptional regulation in early *Drosophila* development via genomic regulatory regions (enhancers), we must first have robust experimental information on the location and affinity of the TF binding sites (TFBSs) present within an enhancer region. For over four decades, computational biologists have worked on developing and refining computational algorithms to predict DNA binding sites. Pioneering studies by Berg and von Hippel in the late 1980s [60–63] established a conceptual understanding of the structural, thermodynamic and statistical rules that impact the binding of TFs to functional sites in DNA. In addition to providing a theoretical framework that enables us to move from qualitative data, in the form of a collection of identified binding sites without affinity measurements, to robust quantitative models that fit the data well [61–63]. One of the most popular bioinformatic approaches to evolve from these early studies for predicting TFBSs is a Position Weight Matrix (PWM)-based approach that scans a DNA sequence for binding sites of a particular TF of interest (Fig. 5) [63–65]. In general, all PWM-based algorithms score sub-sequences (i.e. potential TFBSs) in the DNA region considered and these scores are often interpreted as being proportional to the binding affinity of the sequence. However, the accuracy of the quantitative models generated in PWMs may depend on the quality, and perhaps quantity, of the data used to generate them. As a result, conducting detailed experimental binding assays is important to validate this interpretation. Therefore, the field would benefit from a more comprehensive synergistic approach that would not only integrate existing computational and biochemical methods, but also incorporate more advanced quantitative experimental tools to directly assess the biophysical interaction of HD TFs with DNA binding sequences. This system-level approach would undoubtedly make a valuable contribution by expanding our understanding of the intricate relationship of structure and function in HD-DNA complex formation.

## 2. Established experimental methods to determine protein-DNA binding

Numerous methods have been developed to investigate protein-DNA binding affinity and sequence specificity. In this section, we will discuss the strengths and inherent short-comings of these experimental approaches to examine the binding parameters of a protein-DNA complex.

### 2.1. DNA footprinting

**Fig. 6.**
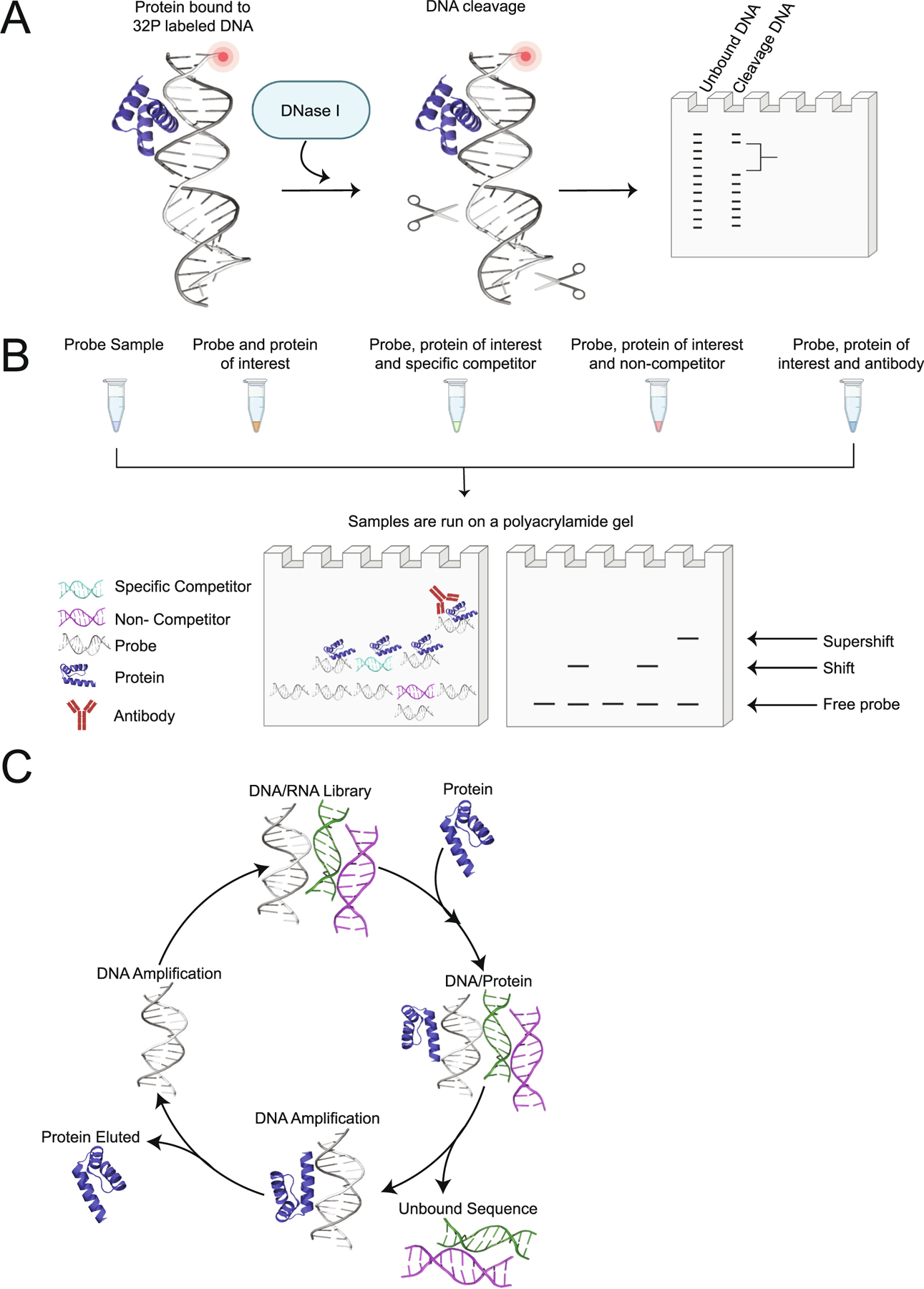
Qualitative methods used to identify transcription factor(TF)-DNA binding sites. (A) DNase I footprinting. After incubating radioactive ^32^P-labeled DNA with a TF, the DNA is cleaved by DNase I. Any protein bound to DNA is considered “protected” from nuclease digestion. The “protected” DNA (aka the DNA footprint) indicates the protein binding site in the DNA sequence. Samples are run on a polyacrylamide gel to visualize TF-DNA binding site [78,168]. (B) Electrophoretic Mobility Shift Assay (EMSA). Radio-labeled DNA and the protein of interest are combined and incubated, then run on a polyacrylamide gel. Electrophoretic mobility changes indicate that a protein-DNA complex has formed. A supershift can be observed when a protein specific antibody is also bound to protein-DNA complex [81]. (C) Systematic Evolution of Ligands by Exponential Enrichment (SELEX). An oligonucleotide library consisting of randomly generated sequences are exposed to a target protein. Any unbound sequences or proteins are removed and only bound sequences are amplified by DNA polymerase. The amplified sequences are used for subsequent rounds of selection, which are then increased to identify the tightest binding sequence [87]. Protein structures (PDB 1JGG) [19] were visualized using Pymol [167] and each schematic was created with BioRender.com.

DNA footprinting was first described in 1978 by Galas and Schmitz [66] and quickly became a commonly used method to determine the sequence specificity of a protein bound to DNA. This approach is based on the principle that any DNA bound by a protein is protected from enzyme cleavage (i.e. nucleases) and/or chemical hydrolysis (i.e. acid/base), while any unprotected DNA will be cleaved (Fig. 6a).

Deoxyribonuclease I (DNase I) is normally used in DNA footprinting experiments because of its large size and ability to function as a single-stranded or double-stranded endonuclease [67]. DNase I binds to the minor groove of DNA and cleaves the phosphodiester backbone producing shorter oligonucleotides. However, DNase I does not cleave uniformly along the DNA [67], due to the inconsistent cleavage rate of the enzyme that is dependent on the local and global fold of the DNA [68] and other factors such as DNA flexibility and sequence differentiation in the minor groove of DNA. When DNase I binds to the oligonucleotide, the DNA becomes dramatically distorted as the minor groove widens and the DNA is bent towards the major groove, eventually allowing for DNA cleavage to occur [69]. Other treatments used in DNA footprinting experiments include hydroxyl radicals, ultraviolet irradiation, and dimethyl sulfate [70]. Hydroxyl radicals are formed by reducing iron(II) with hydrogen peroxide, which then interact with the DNA backbone resulting in random single base deletions [71]. Hydroxyl radicals are not sequence dependent and result in an evenly distributed ladder, but the reaction and digestion time is slow [70]. Ultraviolet irradiation causes the nucleic acid bases to become excited and undergo photoreactions, such as thymine dimerization [72]. Photoreactions are sensitive to the local environment of the DNA and if a protein is bound to the DNA, the environment is altered thereby generating a footprint [72]. Ultraviolet irradiation is a good cleavage agent because it reacts quickly and can penetrate through cell membranes, but the footprinting signal can be unpredictable [72]. Dimethyl sulfate is also a commonly used footprinting reagent that methylates purines in the major groove when not protected by a bound protein. However, this reagent requires extended incubation with the sample that can allow for the dimethyl sulfate to potentially react with the protein, which in turn can result in unwanted DNA footprinting artifacts [72].

Many protein-DNA interaction sites, and the biological function of such interactions, have been determined using this approach. For example, Ekker et al. examined the ULTRABITHORAX (UBX) HD TF, which is responsible for the proper developmental differentiation of wings and halteres in *Drosophila* [73]. The researchers observed that mutations in the HD of UBX resulted in the transformation of the haltere tissue into the wing tissue [73]. In this study, they were also able to determine that UBX recognizes a consensus sequence of 5′-TTAATGG-3′, with the 5′-TAAT′−3′ core playing a large role in determining the binding affinity of the protein, while the flanking bases contribute to overall affinity [74]. Although DNase I footprinting was able to experimentally determine this 7 bp binding site for UBX, the authors noted that naturally occurring UBX binding sites in the genome are typically 40–90 bp and consist of multiple TAA tandem repeats [74]. In parallel studies, Han et al. were able to identify nine protein binding sites in the proximal enhancer that can regulate the expression of the *ftz* gene using DNA footprinting, with each binding site containing the sequence 5′-AGGA-3′ [75]. Wang et al. demonstrated that VENTRAL NERVOUS SYSTEM DEFECTIVE (VND)/NK-2 - HD binds to a purified genomic DNA fragment at four distinct sites, with three sites being strongly protected (containing a 5′-AAGTG-3′ core sequence) and another site that was weakly protected (5′-CAGAGTTT 3′) [76].

Although this approach has uncovered numerous HD binding sites, DNase I footprinting is a very low-throughput method that can only analyze a relatively small region (<1 kb) at a time [77]. Limitations to the DNA footprinting approach include the need for the DNA sequence to be radio-labeled as well as an excess of DNA-binding activity over the amount of DNA fragment used to ensure a clear footprint [78]. Unfortunately, DNA footprinting also cannot distinguish individual components of heterogeneous protein-DNA complexes. As DNase I does not cleave DNA indiscriminately, this can result in the cleaved DNA running unevenly on a polyacrylamide gel that can make it difficult to delineate where the protein provides protection to the DNA (i.e. binding site(s)) [78]. To ensure appropriate digestion, the concentration of DNase I added should be optimized, as DNase I overdigestion can result in faint bands at the top of the probe, more intense bands at the bottom, and an uneven DNA fragment ladder [79]. Another drawback of DNA footprinting is that the yield of the protected fragment may be small and hard to observe if the binding constant for the specific sequence is not larger than the binding constant of the DNA [66]. Due to these limitations, more modern approaches have been implemented to study the interactions between TF HDs and DNA.

### 2.2. Electrophoretic Mobility Shift Assays (EMSAs)

The Electrophoretic Mobility Shift Assay (EMSA) is a useful method that has been extensively used to examine and verify TF binding to different DNA sequences. EMSA is a qualitative approach that utilizes the fact that protein bound to DNA is less mobile than free DNA when migrating through a gel matrix [80]. The binding of a protein to DNA results in an upward shift on the gel due to changes in size and charge of the protein-DNA complex that is formed (Fig. 6b). The migration of DNA in an EMSA is traditionally monitored through the use of radioisotope-labeled nucleic acids [81].

For example, in 2012 Anderson et al. examined the HD TF MOHAWK (MKX), a key regulator of skeletal muscle and tendon differentiation that specifically binds to an inverted repeat 5′-ACAN_25_TGT-3′ core sequence separated by 25 bases [82]. EMSAs were also performed on *D. melanogaster* HD-TFs FTZ, ANTP, ABDOMINAL-A (ABD-A) and ABDOMINAL-B (ABD-B) in our own earlier studies to confirm that their isolated HDs were biochemically functional due to their ability to bind specifically to the consensus sequence containing the 5′-TAAT-3′ core [83]. Although EMSAs can be adapted to any protein, the disadvantages include the fact that the samples are not at chemical equilibrium during the electrophoresis step, and that the rapid dissociation of the protein-DNA complex can potentially hinder detection [81], and the need for the DNA sequence being tested to be radio-labeled (i.e. ^32^P) or fluorescently tagged at the 5′ and/or 3′ end for visualization in classical EMSA assays.

### 2.3. Systematic evolution of ligands by exponential enrichment (SELEX)

Systematic evolution of ligands by exponential enrichment (SELEX) is a method that has been used to reveal DNA binding profiles of numerous TFs. This method isolates specific nucleotide sequences that bind to TF proteins through a repetitive enrichment process that include the elution of bound oligonucleotides [84], followed by amplification using a DNA polymerase (Fig. 6c).

A prime example of using SELEX to determine TF binding sequences was performed by Slattery et al. in 2011, where eight *Drosophila* TFs (LABIAL (LAB), PROBOSCIPEDIA (PB), DEFORMED (DFD), SEX COMBS REDUCED (SCR), ANTP, ABD-A, ABD-B, and UBX) were examined in complex with EXD [4]. Intriguingly, all eight HD TFs bound to similar sequences in vitro; however, when the EXD cofactor was present, each TF-HD tested bound to the DNA sequence with improved specificity [4,85]. For example, while both EXD-SCR and EXD-UBX were observed to bind to 5′-TGATTTAT-3′, EXD-SCR binds more strongly to 5′-TGA-CAAAT-3′ and 5′-TGATTAAC-3′, while EXD-UBX binds more strongly to 5′-TGATTTAC-3′ [4]. This finding shows that each HD TF has distinct binding preferences that are revealed when the EXD cofactor is present, thereby unlocking latent specificities that are present within the protein-DNA complex formed [4].

While this approach is a high-throughput technique to identify DNA binding sequences, a potential drawback of using SELEX is that it relies on the use of a randomized DNA library. The size of the randomized library defines the libraries diversity, where the smaller the library the lower the chance that the strong binding sites are present in the library [86]. The first two rounds of selection cover a wide range of affinities and the following rounds are biased towards a higher affinity sequence motif, which can lead to unintended sequence selection bias due to multiple rounds of enrichment, elution, and amplification [87]. Confirming SELEX experiments using additional protein-DNA approaches is critically important to insure reproducibility and validate results [4]. Recent advances to this approach have been made by using No Read Left Behind (NRLB) in combination with SELEX [88]. NRLB is a model developed by Rastogi et al. than can be used to predict and identify binding affinities through SELEX data analysis [88]. This model only uses a single round of SELEX data to properly predict protein-DNA binding sites and the input data consists of read out sequences from both “round zero” (R0) and after one cycle (R1) [88]. Rastogi et al. successfully implemented this approach to further investigate the binding specificity and detect binding sites of *D. melanogaster* Hox proteins in the presence of the cofactor EXD [88]. Although this approach has been used to infer binding affinities of TFs more accurately than SELEX alone and has been employed to assess both Protein Binding Microarrays (PBMs) and Chromatin immunoprecipitation-sequencing (ChIP-seq; please see next section) [89], further investigation into more TF HDs is required.

### 2.4. Chromatin immunoprecipitation-sequencing (ChIP-seq)

**Fig. 7.**
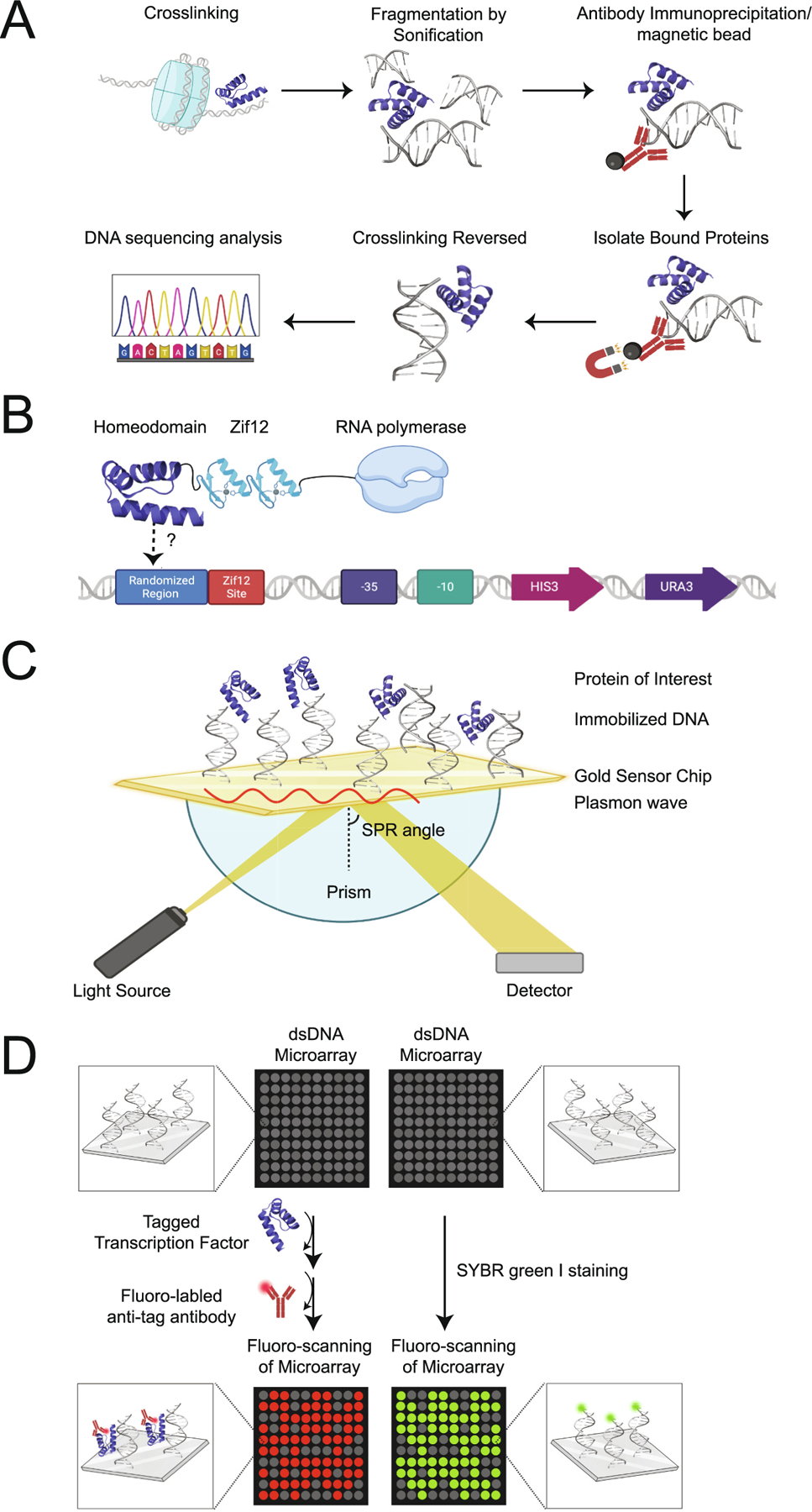
Advanced qualitative methods used to determine transcription factor(TF)-DNA binding sites. (A). Chromatin ImmunoPrecipitation-sequencing (ChIP-seq). Chromosomal DNA crosslinked to a protein is fragmented by sonication. An antibody specific to the protein coupled with a magnetic bead is used to isolate the bound protein-DNA fragments. The chemical crosslinking is then reversed using SDS and the released DNA sequence is analyzed [94]. (B) Bacterial One-Hybrid (B1H). A randomized DNA library representing all potential transcription factor binding sites is cloned immediately upstream from the selectable markers, *HIS3* and *URA3*. If a TF binds to the randomized nucleotide region, the fused RNA polymerase will be recruited to the downstream weak promoter, enabling transcription to occur. Sequences that demonstrate *HIS3* and *URA3* selection are determined to be TF-DNA binding sites [104]. (C) Surface Plasmon Resonance (SPR). An incident light beam is passed through a prism and is reflected off a sensor chip surface containing an immobilized ligand (i.e. DNA fragment). The light is reflected off the surface into a detector. At a certain incident angle (aka resonance angle), light is absorbed by electrons (aka surface plasmons) in the sensor chip causing them to resonate. As molecular binding event occurs, the angular position of light changes [116]. (D) Protein Binding Microarrays (PBMs). A DNA binding protein is expressed and purified containing an epitope tag. The tagged protein is applied to a dsDNA microarray and the microarray is washed to remove any nonspecific protein [120,121]. A fluorescently labeled antibody is then used to quantify the amount of protein bound to DNA. The amount of protein bound to each probe sequence on the array can be quantified [119,121]. Separate microarrays are stained with a dye, SYBR Green I, specific to double-stranded DNA to normalize the PBM data by relative DNA concentrations present in each spot on the microarray [169]. Protein structures (PDB 1JGG) [19] were visualized using Pymol [167] and each schematic was created with BioRender.com. (For interpretation of the references to colour in this figure legend, the reader is referred to the web version of this article.)

ChIP-seq is another qualitative approach that has been widely used to examine TF-DNA binding [90,91]. This method was initially developed by Albert et al. in 2007 to sequence the DNA of 322,000 individual *Saccharomyces cerevisiae* nucleosomes containing a histone variant [92], and can be used to rapidly decode millions of DNA fragments simultaneously, revealing the specific sequences of the precipitated DNA fragments (Fig. 7a) [93]. The ChIP-seq assay is initiated when formaldehyde is added to crosslink the protein-DNA complex, followed by chromatin shearing into small fragments by sonication. A protein specific antibody is then used to immunoprecipiate the protein-DNA complexes and the crosslinking is reversed using sodium dodecyl sulfate to denature the bound protein. The final step is to extract the isolated DNA fragments, representing the TF-bound sequences, and perform DNA sequencing [94].

ChIP-seq has been employed extensively in the last decade to provide maps for various TF binding sites across the entire genome. For example, Jusiak et al. used ChIP-seq to map the binding regions for the SINE OCULUS (SO), a HD TF that is necessary for *Drosophila* eye development [95]. The authors were able to identify 7566 SO enriched DNA regions that mapped to 5952 genes [95]. Although ChIP-seq can identify the genomic regions bound by a target TF, it requires a high-quality antibody for each TF being studied to ensure the detection of enrichment peaks without considerable background noise [96]. A potential limitation to using ChIP-seq is that it is generally qualitative in terms of the sequences that it extracts and it is not possible to distinguish binding affinities between two different complexes of the same TF [85,97]. Because TFs can bind to a variety of genomic loci with different patterns of chromatin markers and nucleosomal organization, this approach might not just reveal TF DNA binding sites, but also locations of modified histones, and binding between other TFs and nucleosomes [98,99]. Another drawback to ChIP-seq is not having sufficient coverage of the particular genome being sequenced. In the case of *Drosophila*, even if a ChIP-seq experiment results in >8 million reads, which is generally considered as sufficient coverage for the genome, there is still a high likelihood that some strong TF binding sites could be overlooked [98,100]. Other limitations of this approach include the requirement of a large amount of sample and the importance of having antibodies that offer high specificity and sensitivity needed to obtain a successful result [96,101]. Lastly, with the use of formaldehyde, ChIP-seq is generally considered a non-native approach, but recent advances are shifting towards using more native-like procedures that does not require chemical cross-linking [102] with promising results showing higher immunoprecipitation efficiency [103].

### 2.5. Bacterial One Hybrid (B1H)

Bacterial One Hybrid (B1H) systems have been used to identify and verify DNA-binding specificity for numerous HD TFs in *Drosophila* [104,105]. This technique involves three major components – a TF expression vector, a bacterial selection strain, and a randomized binding site library in a reporter vector [106] containing a weak promoter [107]. The DNA library undergoes a round of negative selection on plates containing a 5-fluoroorotic acid (5-FOA), allowing for the reduction of self-activating sequences [108]. Two reporter systems can also be used, typically the yeast *HIS3* and/or *URA3* gene, allowing for a combination of positive and negative selection [109]. Each DNA binding domain is expressed as a fusion to an RNA polymerase subunit [109], so that if the DNA-binding domain binds to the target DNA site in the reporter vector, the RNA polymerase is recruited to the promoter and transcription is activated [109] (Fig. 7b).

In 2008, Noyes et al. used a B1H system as a high-throughput method to characterize and determine the specificity of 35 members of the *Drosophila* segmentation network that play important roles in early anterior-posterior patterning [110]. Their study revealed that using a B1H system better defined the recognition motif, 5′-TTTATG-3′ for CAUDAL (CAD), when compared to SELEX or DNase I footprinting approaches [110]. The B1H model has also been used to confirm differences between species. For example, B1H was used to determine that the preferred recognition sequence of the murine MKX HD TF was 5′-ACA-3′, which was subtly different from the 5-′TnACA′−3′ consensus binding site of *Drosophila* MKX [111].

The B1H system is a rapid and streamlined technique used to characterize DNA-binding specificity that can be used to investigate protein-DNA interactions with a DNA library [109]. Although several binding site sequences have been identified for several TFs using the B1H system [109], a drawback to this method is that it only provides qualitative information on a TF-DNA complex. Another limitation is the binding site (s) might not be recognized for TFs with low binding affinity because of competition created by alternative binding sites elsewhere in the bacterial genome limiting the signal generated from a single low affinity binding site upstream of the reporter [108,112].

### 2.6. Surface Plasmon Resonance (SPR)

Surface Plasmon Resonance (SPR) is an optical technique used to measure molecular interactions in real time [113,114]. SPR examines can be used to measure the rate of complex formation as well as provide thermodynamic information [115] (Fig. 7c). Protein-ligand and protein-protein interactions have been routinely evaluated using SPR, but protein-DNA interactions have proven to be more difficult using this technique due to higher binding affinities and the contribution of strong electrostatic interactions [116]. For example, Yoo et al. examined the binding of VND, a HD TF that is important for neuroblast formation during the development of the embryonic central nervous sytem in the *Drosophila*, which recognizes a 5′-T (T/C)AAGT(G/A)G-3′ core motif [117,118]. SPR was used to determine the binding affinity and specificity of full length VND (VND-FL) and VND containing a NK2-specific domain (VND-NH), which is located near the *C*-terminus of the protein. The data showed that VND-FL bound to the DNA sequence with a dissociation constant (K_D_) of 1.53 × 10^−8^ M, while VND-NH bound to the same DNA sequence with a K_D_ of 4.56 × 10^−8^ M, meaning the binding affinity of VND-NH 3-fold weaker than that of VND-FL [118].

Limitations of SPR include artifacts that can be created by aberrant changes of the refractive index at the sensing surface, which can interfere with the interpretation of binding data [118]. Another issue with using SPR to measure protein-DNA interactions is having to manage the mass transfer effect, in which the released protein rebinds to another DNA sequence after dissociating [116]. This phenomenon results in a slower dissociation rate measured by SPR, thereby altering the measured binding affinities. Ideally, to determine the binding affinity of protein-DNA using SPR, a previous experimentally determined binding affinity is needed; however, in most cases, an estimated association constant (K_A_) value must be used causing longer SPR experimental optimization.

### 2.7. Protein Binding Microarrays (PBMs)

Protein Binding Microarrays (PBMs) are a high-throughput approach that can used to visualize protein-DNA interactions in vitro [119,120]. This approach allows for the simultaneous characterization and analysis of a protein’s binding specificity to thousands of double-stranded DNA (dsDNA) probes affixed to the surface of a microarray [119]. PBMs involve expressing and purifying a DNA binding protein with an epitope tag. The tagged DNA binding protein is then applied to a dsDNA microarray and the microarray is extensively washed to ensure the removal of any nonspecific bound protein [120,121]. A fluorescently labeled antibody is then used to quantify the amount of protein bound to DNA. The amount of protein bound to each probe sequence on the array can be quantified based upon signal intensity [119,121] (Fig. 7d).

Busser et al. defined the DNA sequences bound by eleven TF HDs by using PBMs – SLOUCH (SLOU), MUSCLE SEGMENT HOMEOBOX (MSH), BAGPIPE (BAP), LADYBIRD LATE (LBL), PITUITARY HOMEOBOX 1 (PTX1), SIX HOMEOBOX 4 (SIX4), TINMAN (TIN), EVEN-SKIPPED (EVE), UBX, and ABD-B [122]. Their PBM results revealed that UBX, ABD-B, SLOU, MSH, EVE and LBL preferentially recognize a binding sequence core of 5′-TAAT-3′, which was in good agreement with other TF HD-DNA binding studies [123]. Although these TF HDs bind primarily to a sequences containing a 5′-TAAT-3′ core, PBM results showed that SLOU and MSH also recognized a small number of atypical and non-consensus sequences that are unique to the TF HD [122]. Other TF HDs such as TIN and BAP were shown that they could be displaced with DNA sequences that were distinct from the typical 5′-TAAT-3′ core sequence altogether [122]. While Busser et al. were able to successfully determine the binding sequences using PBMs, this approach does have some inherent limitations. PBMs are limited by the amount of sequence that can be represented on the microarray as well as the length of the DNA sequences, supporting on average 60 bp long DNA probes [119]. Lastly, the in vitro nature of PBMs can complicate predicting function TF-binding sites in vivo because it is impossible to replicate the in vivo nuclear environment on a microarray surface [119].

### 2.8. Inherent limitations of existing methods

As described in the previous sections, the various methods used to measure TF-DNA affinities have their individual strengths and drawbacks. For example, although ChIP-seq provides high resolution sequencing and has a wide dynamic range, this technique requires multiple rounds of experimental optimization [91]. Likewise, DNA footprinting can provide a fast and real time analysis, but requires a labeled sample, while SELEX requires multiple rounds of selection [109]. Although performing EMSAs are rapid and can be sensitive, the samples are not in chemical equilibrium and the TF-DNA complex may not be stable resulting in dissociation during electrophoresis, thus causing a miscalculation of binding density [81,91]. For example, Matos et al. used *E. coli* RNase II as a model system to compare SPR and EMSAs for characterization and interpretation of the stability of RNA-protein complexes [124]. EMSAs are frequently used in the characterization of RNA binding, but they require a radioactive labeled RNA (usually ^32^P) and use an indirect method to determine the dissociation constant. To overcome this obstacle, the researchers used SPR to determine the binding ability of wildtype RNase II and sixteen RNase II mutants to RNA [124]. After comparing their EMSA and SPR results, the researchers found that they were not in agreement [124]. EMSA requires a highly stable complex in order to be detected by electrophoresis otherwise a smear is visualized on the gel rather than a clear band. Their study concluded that using SPR was faster and more reliable than running EMSAs that can cause issues with reproducibility and sensitivity [124].

Overall, the techniques discussed in this section provide qualitative protein-DNA binding information, and due to their inherent limitations, many of these techniques have been used in various combinations to assess protein-DNA complex formation. For example, Nitta et al. successfully determined the DNA-binding sites for 92 *Drosophila* TFs [125]. The researchers plotted a dendrogram to compare the similarities between SELEX and B1H motif datasets collected and observed broad agreement in the results, with the same core binding specificities detected with very few exceptions. In some cases, the motifs obtained were not found in both datasets of the dendrogram, which was suggested to be caused by the flanking sequences used for the SELEX not being identical for the B1H dataset [107,125].

Although Matos et al. and Nitta et al. used complementary approaches to examine the binding site specificity, most reported TF-DNA studies only use a single method to determine the DNA binding specificity of a TF. While these individual methods can be useful in assessing TF-DNA binding, they are generally qualitative and may provide inadequate information regarding detailed affinity and sequence specificity. Specificity distinguishes specific binding partners from non-specific binding partners and affinity can be defined as the strength of binding [126], while both are defining characteristics they are not always interrelated. For example, mutations affecting specificity can alter the affinity with their binding partners, but mutations altering the overall binding affinity do not always change the binding specifity [127]. In other cases, the binding affinity and specificity for instant DNA-DNA interactions are anticorrelated as the affinity increases the specificity also increases [128]. One can think of the specifity of any TF as a measure of the variance around the mean affinity; thus, TFs with high specificity have a corresponsing high affinity variance, irrespective of the mean affinity value.

Furthermore, most of the current methods simply determine relative binding affinities for each of the DNA sequences considered. However, the scale of relativity is typically non-linear and any score obtained is itself unitless. As a result, one would need more than a single measurement of a particular sequence to determine absolute affinities for all other sequences investigated. In fact, detailed and accurate parameterization, presumably obtained via robust parameter estimation approaches, would be required to determine exactly how the relative scores relate to absolute binding affinity values for all sequeuences examined.

A major question that still needs to be addressed in the field is how do these TFs recognize and bind to their DNA targets and what are the true binding affinities of these TF-DNA interactions? Here, we call for the adoption of more robust and reproducible biophysical measurements of binding affinities to decipher the important roles that TFs play in cell fate and embryonic development in *Drosophila*.

## 3. Advanced quantitative approaches to measure TF-DNA affinities

### 3.1. Computational methods to investigate TF-DNA binding sites

A variety of different computational approaches have been used to further examine the binding affinity of TF-DNA complexes. These approaches have drawn on different areas of computational biology, including sequence alignment, machine learning, and hidden Markov models. To date, the most commonly implemented subset of these approaches are those that involve the construction of PWMs [129,130]. These algorithms begin with a set of known binding sequences for the protein of interest that are typically identified using in vitro approaches such as the B1H system (described in detail above). This list of binding sequences is used to construct a PWM containing the frequency of each of the four possible nucleotides at each position of the binding sequence (Fig. 5). Binding strengths are then inferred for individual proteins and DNA sequences of interest relative to a ‘consensus sequence’, the sequence found to have the most common nucleotide in each position of the binding sequence (Fig. 5). The underlying assumption of this approach is that sequences with the same physical binding affinity are equally likely to be selected by natural selection, thus those that appear in the set of known binding sequences most often represent the sequences with highest affinity, and the calculated binding energies for protein contacts at each individual base pair are additive (i.e. nucleotide positions within a binding sequence are independent of each other) [63,129–133]. These approaches can quickly score the strength of a large number of potential binding sequences for a protein of interest based on the PWM and nucleotide genomic background frequency [129,130]. For this reason, these methods have been widely used in the discovery of *cis*-regulatory elements, including enhancers, as well as the identification of TF binding sites used in mathematical modeling studies of transcriptional regulation.

One such study implemented a modified version of the PWM-based program Patser, which allowed the authors to search for potential TF binding sites in the putative *cis*-regulatory elements identified by ChIP-seq analysis [134]. The authors were able to identify potential binding sites for the homeobox TFs SCR, ANTP, and UBX, which are known to regulate the expression of additional genes within the HOM-C complex of *Drosophila* [134]. A similar study by Ostrin et al. used a PWM-based algorithm to predict potential binding sites for the TF EYELESS (EY) within the genome of *D. melanogaster* [135]. This allowed the authors to identify potential EY binding sites and prioritize a subset of target genes for further functional analysis [135]. More recently, increasingly complex models have been developed that take into account the non-independence of positions within a binding site [136–138]. Such models allow for the development of PWMs that have been shown in some scenarios to quantitatively outperform a standard PWM in terms of predictive ability [139].

In addition to TF binding site discovery, many mathematical modeling studies would not be possible without the identification of putative TF binding sites through bioinformatic algorithms. Thermodynamic-based models of transcriptional regulation in *Drosophila* have relied heavily on PWM-based methods over the last two decades [47–59]. These models focus on predicting the expression of a gene based entirely on the sequence of a corresponding enhancer, or *cis*-regulatory module. For example, a recent study by Bhogale et al. employed thermodynamic-based models (an extension of GEMSTAT [50]) to explore the role of indirect binding of TFs during *Drosophila* embryogenesis and gain insights into how a TF may switch between transcriptional activation and repression roles [59]. The first step in their modeling process was to use a PWM-based algorithm to estimate the binding strengths of all putative sites within each enhancer they analyzed. This was a crucial first step, as the GEMSTAT model relies on these putative binding sites to calculate the predicted target gene expression. They then investigated the role of indirect binding of TFs by allowing TFs to “piggyback” on other bound TFs to modulate the overall impact on gene expression. The study found that by allowing this indirect binding of TFs to influence the model output, their model was able to more accurately predict gene output during *Drosophila* mesoderm development. This suggests that TF-TF interactions, where both TFs are not directly bound to the enhancer, may underlie the dual regulatory role observed for some TFs [59]. Although studies such as this demonstrate the usefulness of PWM-based approaches in understanding the regulatory mechanisms underlying *Drosophila* embryonic development, using PWM-based scores as a proxy for the true binding affinity of a TF for a DNA sequence could be problematic. The assumption made is that there is a direct correlation between binding affinity and the frequency with which we find a particular nucleotide in a given position within the set of known binding sites. To our knowledge, this assumption has never been robustly validated using quantitative biophysical experimental approaches.

Thermodynamic modeling has also been applied to other mammalian systems. For example, Bertolino et al. developed a thermodynamic model which utilizes PWMs to identify binding sites [140]. The researchers were able to identify seven *cis*-regulatory elements neighboring *Cebpa*, a gene encoding for a TF called CCAAT enhancer-binding protein alpha that play a role in the development of myeloid cells [140]. Regardless of the model system, the use of PWMs in combination with experimentally determined binding sites using biophysical approaches can help to decipher where, when, why, and how TFs recognize and bind to different DNA sequences.

### 3.2. Mechanically Induced Trapping of Molecular Interactions (MITOMI)

Mechanically Induced Trapping of Molecular Interactions (MITOMI) is a newer method that can be used to obtain affinity constants and kinetic rate of protein-protein, protein-ligand, and protein-DNA interactions [141,142]. This approach allows for the capture of low affinity interactions between TFs and DNA target sequences by measuring the absolute binding affinities [143,144].

**Fig. 8.**
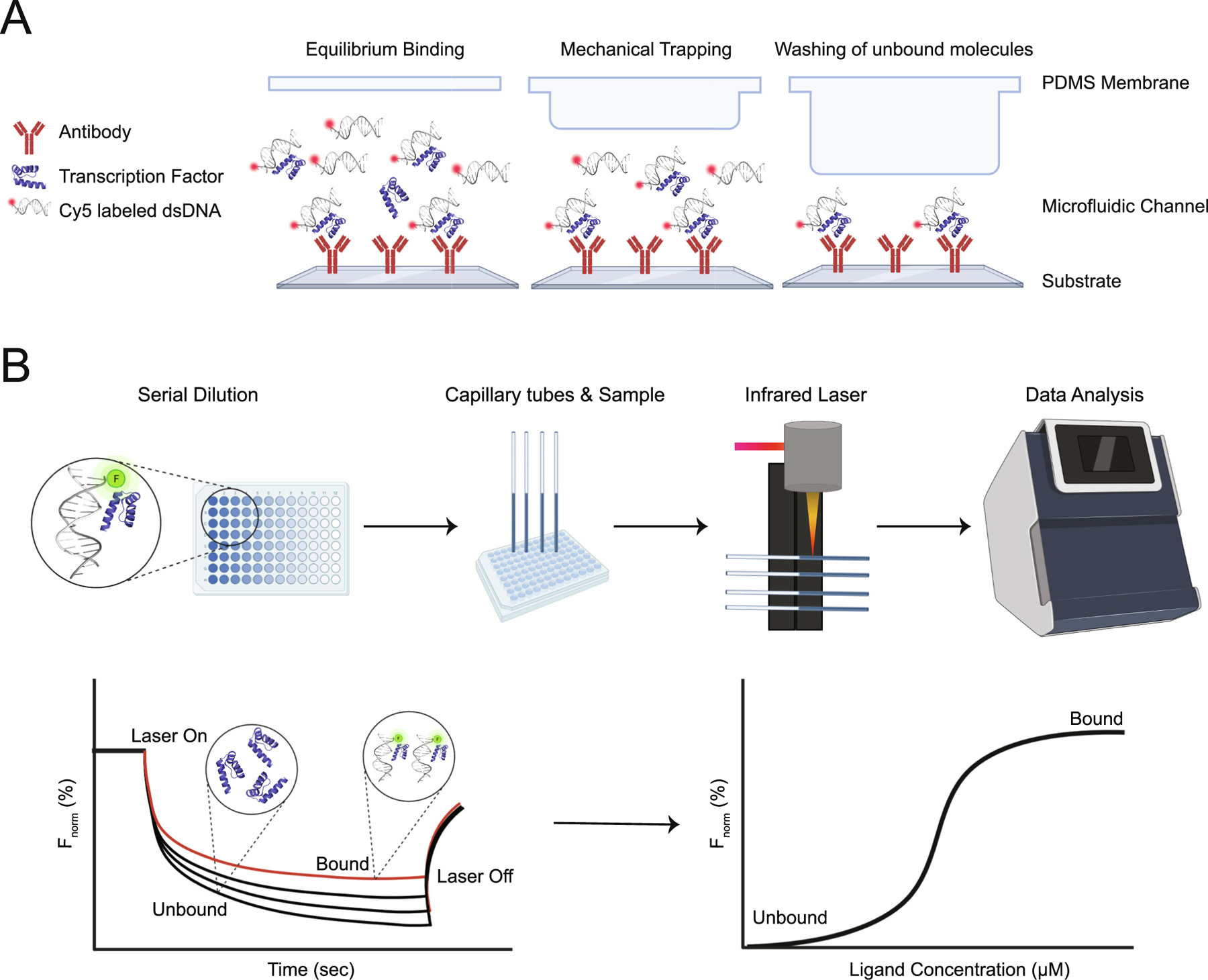
More quantitative methods used to determine affinity and specificity of TF-DNA binding. (A) Mechanically Induced Trapping of Molecular Interactions (MITOMI). During equilibrium binding, the fluorescently labeled ligands bind to a target immobilized on the bottom surface of a membrane [142]. Mechanical trapping occurs when the bottom membrane is actuated, causing the surface-bound complexes to become trapped and expelling the solution phase molecules [142,144]. Unbound molecules are washed away and the trapped material is quantified by signal intensity [142,144]. (B) MicroScale Thermophoresis (MST). MST detects the movement of molecules within a temperature gradient. Binding events are detected by measuring changes in fluorescence intensity with increasing concentration of the ligand. First, the sample is pulled into a capillary tube by surface tension. An Infrared (IR) laser is switched on and unbound/bound molecules move out of the heated spot. A spectral shift is determined by the depletion of fluorescence caused by the slower movement of the bound TF-DNA complex, when compared to the unbound molecules, and then integrated into a titration curve [148]. Protein structures (PDB 1JGG) [19] were visualized using Pymol [167] and each schematic was created with BioRender.com.

This technique employs a microfluidic device to directly measure the binding specifies of TF to numerous DNA sites [144] (Fig. 8A). First, spots of Cy5-labeled dsDNA sequences are printed onto an epoxy-coated microscope glass slide with each DNA sequence spotted with varying concentration to allow for a saturation binding curve and absolute binding affinities to be measured [142]. The DNA arrays are then aligned to a microfluidic chip, containing 768 unit cells [142,143]. Anti-penta histidine antibodies are immobilized under the button membrane and the device is loaded with a wheat germ in vitro transcription/translation (ITT) solution that contains the DNA template coding for the protein [142,143]. The chip is then loaded with the fluorescently labeled protein and binds to the immobilized antibodies. The ITT solution flows into the spotting chamber and the DNA interacts with the protein, the TF-DNA bound complexes are trapped, and any unbound or non-specific DNA is washed away [142,143] (Fig. 8a). A DNA array scanner then visualizes the formed complexes and the amount of protein bound is determined by its fluorescent signal intensity [144]. The detected signals are quantified, plotted, and the binding affinities are determined.

He et al. used PWMs to predict affinity differences of naturally occurring mutations in HUNCHBACK (HB) and BCD binding sites [145], where their PWM scores correctly predicted the affinity change for 21/25 mutations [145]. To evaluate the accuracy of the PWM-based inference, the researchers employed MITOMIs to experimentally measure the binding affinity differences and verified that these combined approaches showed good agreement for naturally occurring mutations in HB and BCD binding sites [145]. Other studies using this approach have examined protein-protein interactions [146] and successfully mapped the binding energy landscape of basic helix-loop-helix TFs [147]. Although MITOMIs allow for the investigation of numerous dynamic molecular interactions [141], when compared to other approaches they are relatively low throughput [142] and requires specialized instrumentation [144].

### 3.3. Microscale Thermophoresis (MST)

MST is a quantitative approach that detects movement caused by a biomolecular binding event within a temperature gradient using changes in fluorescence intensity (Fig. 8b) [148]. This sensitive technique detects changes in size, charge and the hydration shell of a molecule [149] and can measure a wide range of biochemical interactions, including protein-small molecule, protein-drug, protein-liposome, and protein-DNA [150]. In MST, a fluorescently labeled partner is mixed with varying concentrations of a nonfluorescent partner. The mixture is loaded into a capillary tube and a temperature gradient is applied that allows for the movement of the fluorescent molecule across the temperature gradient to be measured (Fig. 8). Any protein bound to DNA will escape the heated region slower than any unbound protein due to the larger molecular size of the complex [151]. Experiments are run using a low sample volume at low nanomolar concentrations and the binding affinity can be determined in 10 min in free solution [149]. MST was recently used to measure the binding affinity of TRANSCRIPTION TERMINATION FACTOR I (TTF-1), a transcription factor involved in the termination phase of the transcription cycle, with various DNA sequences [148]. A clear strength of MST is the ability to quickly and reproducibly measure protein-DNA interactions in solution. However, the technique is limited in that it does not provide any information on the thermodynamic properties of the interaction (i.e. enthalpy and entropy).

### 3.4. Isothermal Titration Calorimetry (ITC)

**Fig. 9.**
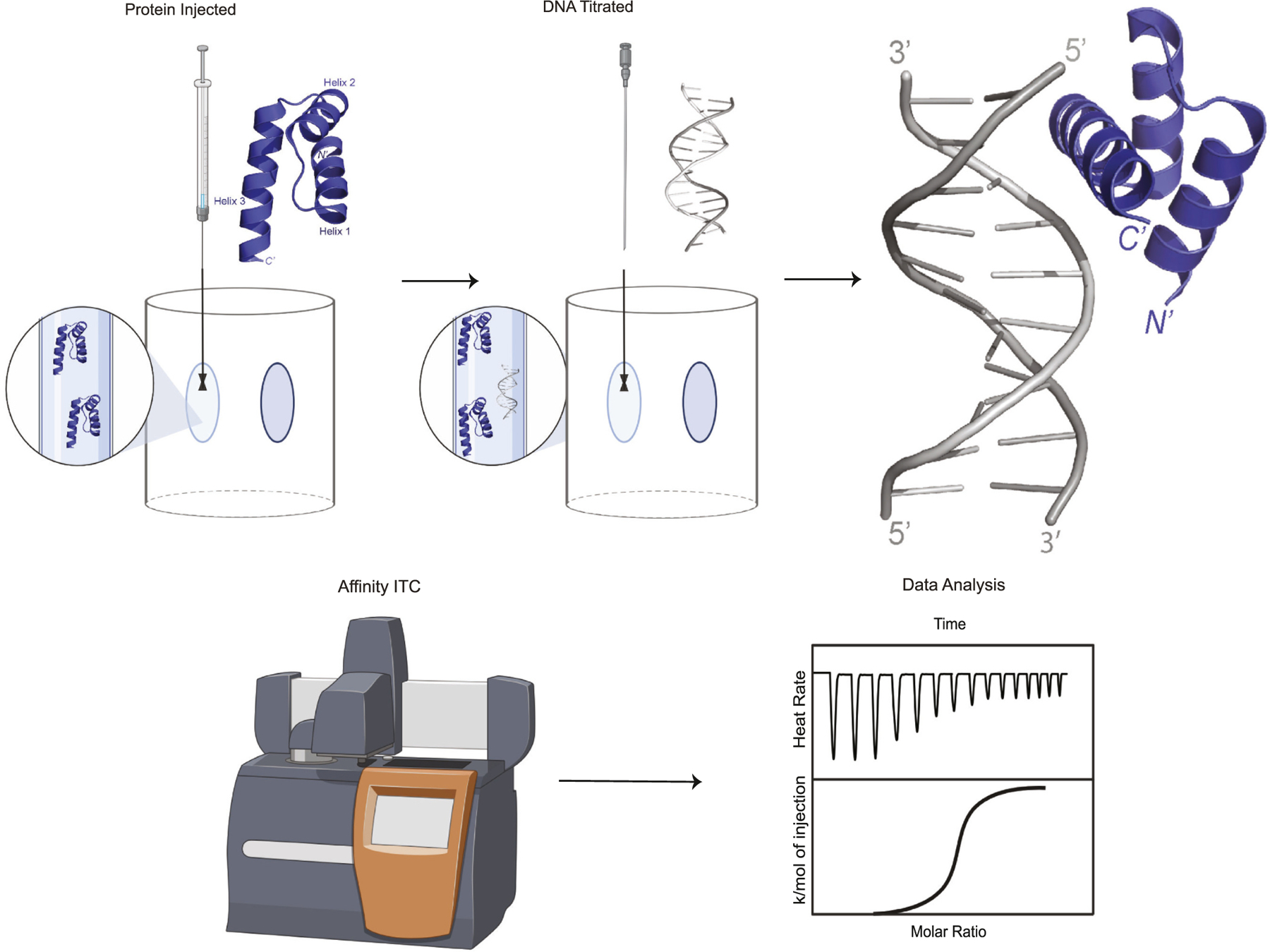
Isothermal Titration Calorimetry (ITC) is a quantitative biophysical method that can determine affinity and specificity of TF-DNA binding. ITC is a robust method that can experimentally determine binding affinity, specificity, and all of the thermodynamic properties involved in a biomolecular interaction (i.e. enthalpy, entropy, and Gibbs free energy). The instrument contains two cells (reference cell and sample cell) contained within an adiabatic jacket and the system is kept under constant pressure and volume. Initially, the protein of interest is injected into the sample cell, then the ligand (i.e. DNA sequence) is subsequently titrated into the sample cell in discrete injections volumes until the protein is fully saturated. The heat released or absorbed during binding is measured throughout the run. Isotherms are obtained and then integrated to plot a titration curve. Protein structures (PDB 1JGG) [19] were visualized using Pymol [167] and each schematic was created with BioRender.com.

Isothermal Titration Calorimetry (ITC) is a quantitative biophysical approach that can be used to accurately measure real binding affinities for TF-DNA complexes (Fig. 9). ITC is generally considered to be the “gold standard” for biophysical measurements of a biomolecular interaction as it is the only technique that allows for the determination of all thermodynamic parameters (i.e. binding constant, molar ratio, Gibbs free energy, entropy and enthalpy) all in a single experiment [152,153]. The instrument is sensitive enough to characterize biological macromolecules, protein-protein, protein-DNA, and protein-small molecule interactions, and enzymatic activity [152].

ITC is a powerful technique that is now starting to be employed to measure the binding affinity of specific TFs with different nucleotide sequences. To conduct an ITC experiment, the protein of interest (i.e. TF HD) is inserted into the sample cell and the ligand (i.e. DNA sequence being tested) is loaded into the syringe. This is because it is easier to reach higher concentration with the ligand than the protein [154]. Over the course of 20–30 sequential injections, the ligand is titrated into the sample cell until the protein is fully saturated. The incremental heat changes from injection are accurately measured and an isotherm is obtained [155]. The isotherms are then integrated into a titration curve to yield the binding constant (K), stoichiometry (n), and the heat (i.e. enthalpy) of the binding event while also calculating the entropy of the system [155].

For example, the TF SPECIAL AT-RICH SEQUENCE BINDING PROTEIN-1 (SATB1), a HD TF found in humans that regulates over 1000 genes and controls transcription in various biological processes, such as switching of fetal globin genes, metastasis of breast cancer, and differentiation of embryonic stem cells, has been assessed using ITC [156]. SATB1 contains multiple DNA-binding domains including a HD and two CUT-domain repeats, CUTr1 and CUTr2, that work together to modulate DNA binding specificity [156]. SATB1 binds to matrix attachment regions (MARs) in DNA, which are sequences of DNA where the nuclear matrix attaches, and plays an important role in higher-order chromatin organization [157]. ITC was used to demonstrate that CUTr1 of SATB1 has binding specificity for the MAR sequence 5-TAATA-3′, while the SATB1 HD has an affinity for a variety of DNA binding sites without specificity [156]. The binding of CUTr1, CUTr2, and the HD to a DNA sequence containing two 5′-TAATA-3′ sites was also examined [156]. The change in enthalpy (ΔH) was determined for both the HD and CUTr1 domains to be −20 kJ/mol and −45 kJ/mol, respectively [156], revealing that CUTr1 binds with a tighter affinity to DNA than the HD alone. In contrast, CUTr2 showed no significant heat release, indicating weaker binding than CUTr1 and the HD and overall poor binding to the 5′-TAATA-3′ sites [156].

Traditionally, ITC has been considered a low throughput approach due to longer experimental running times and the need for extensive cleaning steps between each run. However, new improvements to ITC instrumentation have been made to make this approach more high throughput and less labor intensive. TA instruments (New Castle, DE, USA) have recently brought to market fully automated versions of their iTC_200_ and Affinity ITC that can be interfaced with a robotic sample changer, injector, and cleaning station [158]. The Affinity ITC Auto uses an industry-proven 96-well plate liquid handling system and the Auto-iTC_200_ allows for 30–40 titrations per day, with both systems being able to self-clean and run unattended for extended periods of time [158]. ITC is beginning to emerge as a powerful technique in determining the binding specificity and affinity of TF proteins. With the ability of ITC to experimentally determine all of the thermodynamic parameters of a TF-DNA interaction including stoichiometry and dissociation constant (K_D_) ranging from nM to uM range, this approach has the potential to be used to examine many other TFs, including HD TFs, and determine their respective DNA sequence affinities with remarkable reproducibility and accuracy.

### 3.5. Potential limitations of using in vitro biophysical approaches

Recent in vivo and structural studies examining transcription factors binding to DNA have shown that it is difficult to correlate in vitro findings with in vivo approaches. For example, researchers have reported that the affinity of some HD TFs can be altered due to chromatin DNA packing, which in turn can also regulating their interactions with other transcriptional regulators in the cell [159,160]. Likewise, Mann and coworkers have reported that HD TF binding is dependent on the 3D shape of the DNA binding site, and that the HD and DNA can undergo conformational changes that can affect the strength of the interaction [22,161,162]. Since chromatin packing and the 3D shape of DNA are also intricately connected to the actual sequence of the DNA, these studies add an additional level of nuance and complexity that need to be considered when it comes to examining HD-DNA complex formation and their respective affinities. It will be important for researchers in this growing field to recognize this caveat because what is observed using in vitro approaches may not always agree with what is observed using an in vivo system.

## 4. Conclusions

Homeobox genes, and the homeodomain (HD) TF proteins that they encode, are important master regulators of the transcriptional program that controls proper body segmentation formation during early embryonic development. Structural studies using NMR spectroscopy and X-ray crystallography have proven to be instrumental in helping us better understand the molecular structures of isolated HDs, HD-DNA complexes, and HD-HD cofactor-DNA quaternary structures. Numerous qualitative experimental approaches, together with increasingly sophisticated bioinformatic algorithms, have also provided a great deal of insight on TF-DNA binding. A major limitation to these qualitative approaches is that the binding affinity predictions determined from these methods are unitless and can be difficult to reproduce. The modern field of molecular genetics would therefore benefit from the incorporation of more high quality quantitative measurements of HD TF-DNA binding affinities and more robust genome-wide TF binding site predictions.

We therefore recommend that the field shifts towards the adoption of more quantitative approaches, such as ITC, MST, and MITOMI, to analyze the binding affinity and specificity of these HD TF-DNA complexes. These biophysical measurements can then be combined with computational approaches, such as PWM-based approaches, in an iterative manner to improve future predictions on where, when, and how HD TFs are likely to bind in a eukaryotic genome. The use of an integrated system-level approach, that incorporates more biophysical approaches, will be critical to continue to improve and expand our understanding on how these important TFs function in the molecular control of gene expression during embryonic development.

## Supplementary Material

MMC1

## Figures and Tables

**Table 1:** Solved structures of *D. melanogaster* Homeodomain Transcription Factors.

Homeodomain	PDB/BMRB code	Structural determination method	Citation
ABDOMINAL A	8F37/31057	NMR	^a^
ABDOMINAL B/EXTRADENTICLE	5ZJQ, 5ZJR, 5ZJS, 5ZJT	X-ray	[22]
ANTENNAPEDIA	9ANT, 4XID	X-ray	[23,24]
	1HOM, 1AHD, 1SAN/1037, 4104	NMR	[25–27]
ARISTALESS/CLAWLESS	3A01, 3A02, 3LNQ	X-ray	[28]
	8F36/31056	NMR	^a^
BICOID	1ZQ3/6906	NMR	[21]
EVEN-SKIPPED	1JGG	X-ray	[19]
ENGRAILED	1HDD, 1ENH, 3HDD	X-ray	[29–31]
	2P81, 2JWT, 7YB4/7386, 15536	NMR	[32,33]^a^
FUSHI TARAZU	1FTZ	NMR	[20]
SEX COMBS REDUCED/EXTRADENTICLE	2R5Y, 2R5Z	X-ray	[34]
UBX-EXD-DNA	1B8I, 4CYC, 4UUS	X-ray	[35,36]
VENTRAL NERVOUS SYSTEM/NK-2	1NK2, 1NK3, 1VND/4141	NMR	[37,38]

Recent advances in machine based learning models, such as AlphaFold2, have also opened new avenues to predict the 3D structures of proteins [39] that can be complementary to conventional structural determination methods like NMR and X-ray crystallography. However, it is important to note the caveat that Alpha-Fold2 is a valuable tool for predicting a protein model but will likely not replace experimentally determined structural approaches that can shed more detailed light on the subtle differences in conformation states experienced by each HD protein [40].

a To be published on the Protein Data Bank (PDB) https://www.rcsb.org

## Data Availability

No data was used for the research described in the article.

## Abbreviations^1^

5-FOA: 5-fluoroorotic acid
ABD-A: ABDOMINAL-A*
ABD-B: ABDOMINAL-B*
ANTP: ANTENNAPEDIA*
B1H: bacterial one hybrid
BAP: BAGPIPE*
BCD: BICOID*
CAD: CAUDAL*
Cebpa: CCAAT enhancer-binding protein alpha
ChIP-seq: chromatin immunoprecipitation-sequencing
DFD: DEFORMED*
DNase I: deoxyribonuclease I
KA: association constant
KD: dissociation constant
EMSA: Electrophoretic Mobility Shift Assays
EVE: EVEN-SKIPPED*
EXD: EXTRADENTICLE*
EY: EYELESS*
FTZ: FUSHI TARAZU*
hb: hunchback
HB: HUNCHBACK*
HD: homeodomain
ITC: isothermal titration calorimetry
ITT: in vitro transcription/translation
LAB: LABIAL*
LBL: LADYBIRD LATE*
MARs: matrix attachment regions
MITOMI: mechanically induced trapping of molecular interactions
Mkx: Mohawk
MKX: MOHAWK*
MSH: MUSCLE SPECIFIC HOMEOBOX*
MST: Microscale Thermophoresis
NMR: nuclear magnetic resonance
NRLB: no read left behind
PB: PROBOSCIPEDIA*
PBM: protein binding microarray
PTX1: PITUITARY HOMEOBOX 1*
PWM: position weight matrix
SATB1: SPECIAL AT-RICH SEQUENCE BINDING PROTEIN-1*
SCR: SEX COMBS REDUCED*
SIX4: SIX HOMEOBOX 4*
SLOU: SLOUCH*
SO: SINE OCULUS*
SPR: surface plasmon resonance
SELEX: Systematic Evolution of Ligands by Exponential Enrichment
TFBSs: TF binding sites
TFs: transcription factors
TIN: TINMAN*
TTF-1: TRANSCRIPTION TERMINATION FACTOR I*
UBX: ULTRABITHORAX*
VND-FL: full length VND
VND-NH: VND containing NK2-specific domain
VND/NK: ventral nervous system defective
