# Supplementary material for: Experimental approaches to investigate biophysical interactions between homeodomain transcription factors and DNA
Source: Biochim Biophys Acta Gene Regul Mech. Author manuscript; Available in PMC 2025 Mar 1. (PMC11832328; doi:10.1016/j.bbagrm.2024.195074)
Supplement: MMC1 [file NIHMS2042786-supplement-MMC1.docx]

**Supplemental Tables**

**Table S1.** Solved 3D structures of Eukaryotic Transcription Factor Homeodomains

| Species | Homeodomain | PDB/BMRB code | Structural Determination  Method | Citation |
| --- | --- | --- | --- | --- |
| *A. thaliana* | Hypothetical protein F22K18.140 | 1WH7/10045 | NMR | * |
|  | LUX | 6QEC | X-ray | ^170^ |
|  | WUS | 6RY3, 6RYD, 6RYI, 6RYL | X-ray | ^171^ |
|  | ZF-HD | 1WH5/10044 | NMR | * |
| *C. elegans* | CEH37 | 2MGQ/19600 | NMR | * |
| *D. rerio* | Terfa | 7C4Q, 7C4R, 7ELK | X-ray & NMR | * |
| *G. gallus* | Engrailed | 3ZOB/19049 | NMR | ^172^ |
| *H. sapiens* | ALX4 | 2M0C/18805 | NMR | * |
|  | ATBF1 | 2DA1, 2DA2, 2DA3/10283, 10284, 10285 | NMR | * |
|  | B13 | 2CRA/** | NMR | * |
|  | BarHL1 | 2DMT/10295 | NMR | * |
|  | BARHL2 | 8PN4, 8PNA, 8PNC, 8PM5, 8PM7, 8PMC, 8PMF, 8PMN | X-ray | * |
|  | Brn-5 | 3D1N | X-ray | ^173^ |
|  | CDX1 | 5LUX, 7Q3O | X-ray | ^174^ |
|  | CDX2 | 5LTY, 6ES2, 6ES3 | X-ray | ^174,175^ |
|  | CRX | 9B8U | X-ray | ^176^ |
|  | DLX3 | 4XRS | X-ray | ^177^ |
|  | DLX5 | 2DJN, 4RDU/10290 | X-ray & NMR | * |
|  | DNAJC1 | 2CQQ/** | NMR | * |
|  | DUX4 | 5Z2S, 5Z2T, 5Z6Z, 6A8R, 6E8C, 6U81, 6U82 | X-ray | ^178-182^ |
|  | GBX1 | 2N8G, 2M34, 2ME0, 2ME6/18944, 19511, 25849 | NMR | * |
|  | goosecoid | 2DMU/10296 | NMR | * |
|  | hDMP1 | 2LLK/18051 | NMR | * |
|  | HNF-1 beta | 2DA6/10288 | NMR | * |
|  | Homez | 2ECC, 2YS9/10047, 10298 | NMR | * |
|  | HOX11L1 | 3A03 | X-ray | ^28^ |
|  | HOXA13 | 2L7Z/16252 | NMR | ^183^ |
|  | HOXB1 | 1B72 | X-ray | ^184^ |
|  | HOXB13 | 5EDN, 5EEA, 5EF6, 5NO6, 7PSX, 8BYX | X-ray | ^174,175^ * |
|  | HOXC9 | 2MSY/25142 | NMR | ^185^ |
|  | HTFF1 | 1BA5/4210 | NMR | ^186^ |
|  | hTRF1 | 1W0T | X-ray | ^187^ |
|  | hTRF2 | 1W0U | X-ray | ^187^ |
|  | KIAA1034 | 1WI3/** | NMR | * |
|  | KIAA1903 | 1WGX/** | NMR | * |
|  | KIAA1915 | 2CU7/11322 | NMR | ^188^ |
|  | LCoR | 2COB | X-ray | * |
|  | LHX4 | 5HOD | X-ray | ^174^ |
|  | LIM | 2DMQ/10293 | NMR | * |
|  | LMX1a | 8IK5, 8ILW | X-ray | * |
|  | MEIS1 | 4XRS, 5BNG | X-ray | ^177^ |
|  | MEIS2 | 3K2A | X-ray | * |
|  | Nanog | 2KT0, 2VI6, 4RBO/16680 | X-ray & NMR | ^189,190^ * |
|  | NKX2.5 | 3RKQ | X-ray | ^191^ |
|  | NKX3.1 | 2L9R/17484 | NMR | * |
|  | OCT1 | 1GT0, 1HF0, 1POG, 1OCT, 1O4X/** | X-ray & NMR | ^192-196^ |
|  | OCT2 | 1HDP, 9DZM/** | X-ray & NMR | ^197^ * |
|  | PAX3 | 3CMY | X-ray | ^198^ |
|  | PAX6 | 2CUE, 6PAX/10281 | X-ray & NMR | ^199^ * |
|  | PBX1 | 1PUF | X-ray | ^200^ |
|  | PHOX2B | 8P7G/34822 | NMR | ^201^ |
|  | PIT1 | 5WC9 | X-ray | ^202^ |
|  | PITX2 | 2L7F, 2LKX/17147, 18015 | NMR | ^203^ * |
|  | PKNOX1 | 1X2N/** | NMR | * |
|  | PRH | 2E1O/** | NMR | * |
|  | SATB1 | 2MW8/25307 | NMR | * |
|  | SOX2 | 1O4X/** | NMR | ^195^ |
|  | TEF1 | 2HZD/** | NMR | ^204^ |
|  | TEF3 | 5NO6 | X-ray | * |
|  | TGIF1 | 6FQP | X-ray | ^205^ |
|  | TGIF2LX | 2DMN/10291 | NMR | * |
|  | TRF2 | 3SJM | X-ray | * |
|  | ZHX1 | 2ECB, 2LY9, 3NAR/18714 | X-ray & NMR | ^206^ * |
|  | ZZZ3 | 2YUM/10193 | NMR | * |
| *M. auratus* | PDX1 | 2H1K | X-ray | ^207^ |
| *M. musculus* | GSX2 | 8EML | X-ray | ^208^ |
|  | HOP | 1UHS, 2HI3/** | NMR | ^209^ * |
|  | HOXA9 | 1PUF | X-ray | ^200^ |
|  | HOXA13 | 2LD5/16577 | NMR | ^183^ |
|  | LAG1 | 1X2M/** | NMR | * |
|  | LIM | 2JTN | NMR | ^210^ |
|  | Meis1 | 8VTS | X-ray | ^211^ |
|  | Msx-1 | 1IG7 | X-ray | ^212^ |
|  | Nanog | 2VI6 | X-ray | ^213^ |
|  | OCT3 | 1OCP/** | NMR | ^214^ |
|  | OCT4 | 3L1P | X-ray | ^215^ |
|  | OCT6 | 2XSD | X-ray | ^216^ |
|  | OTX2 | 2DMS/10294 | NMR | * |
|  | PBX1 | 1DU6, 1LFU/4357, 4572 | NMR | ^217,218^ |
|  | RAG1 | 3GNA, 3GNB | X-ray | ^219^ |
|  | RUH-034 | 2CQX/** | NMR | * |
|  | SOX18 | 4Y60 | X-ray | ^220^ |
| *O. anatinus* | sDUX | 8EJO, 8EJP | X-ray | ^221^ |
| *R. norvegicus* | FTT | 1FTT | NMR | ^222^ |
|  | ISL-1 | 1BW5/4121 | NMR | ^223^ |
|  | LFB1 | 1LFB | X-ray | ^224^ |
| *R. rattus* | LFB1 | 2LFB/** | NMR | ^225^ |
| *S. cerevisiae* | MATa1 | 1F43/4637 | NMR | ^226^ |
|  | MATa1/MATa2 | 1AKH, 1K61, 1MNM, 1YRN | X-ray | ^227-230^ |
|  | MATa2 | 1APL | X-ray | ^231^ |

*-to be published on the Protein Data Bank (PDB) https://www.rcsb.org

**-predates the Biological Magnetic Resonance Bank (BMRB) https://bmrb.io

**Table S2.** Solved 3D structures of Prokaryotic Transcription Factor Homeodomains

| Species | Homeodomain | PDB code | Method | Citation |
| --- | --- | --- | --- | --- |
| *E. coli* | MARA | 1BL0 | X-ray | ^232^ |
| *M. truncatula* | STF | 6WIG | X-ray | ^233^ |
| *O. sativa* | PHR2 | 7D3T | X-ray | ^234^ |

**Abbreviations**

| 5-FOA | 5-fluoroorotic acid |
| --- | --- |
| ABD-A | ABDOMINAL-A* |
| ABD-B | ABDOMINAL-B* |
| ANTP | ANTENNAPEDIA* |
| B1H | Bacterial one hybrid |
| BAP | BAGPIPE* |
| BCD | BICOID* |
| CAD | CAUDAL* |
| Cebpa | CCAAT enhancer-binding protein alpha |
| ChIP-seq | Chromatin immunoprecipitation-sequencing |
| DFD | DEFORMED* |
| DNase I | Deoxyribonuclease I |
| KA | Association constant |
| KD | Dissociation constant |
| EMSA | Electrophoretic Mobility Shift Assays |
| EVE | EVEN-SKIPPED* |
| EXD | EXTRADENTICLE* |
| EY | EYELESS* |
| FTZ | FUSHI TARAZU* |
| hb | Hunchback |
| HB | HUNCHBACK* |
| HD | Homeodomain |
| ITC | Isothermal titration calorimetry |
| ITT | in vitro transcription/translation |
| LAB | LABIAL* |
| LBL | LADYBIRD LATE* |
| MARs | Matrix attachment regions |
| MITOMI | Mechanically induced trapping of molecular interactions |
| Mkx | Mohawk |
| MKX | MOHAWK* |
| MSH | MUSCLE SPECIFIC HOMEOBOX* |
| MST | Microscale thermophoresis |
| NMR | Nuclear magnetic resonance |
| NRLB | No read left behind |
| PB | PROBOSCIPEDIA* |
| PBM | Protein binding microarray |
| PTX1 | PITUITARY HOMEOBOX 1* |
| PWM | Position weight matrix |
| SATB1 | SPECIAL AT-RICH SEQUENCE BINDING PROTEIN-1* |
| SCR | SEX COMBS REDUCED* |
| SIX4 | SIX HOMEOBOX 4* |
| SLOU | SLOUCH* |
| SO | SINE OCULUS* |
| SPR | Surface plasmon resonance |
| SELEX | Systematic Evolution of Ligands by Exponential Enrichment |
| TFBSs | TF binding sites |
| TFs | Transcription factors |
| TIN | TINMAN* |
| TTF-1 | TRANSCRIPTION TERMINATION FACTOR I* |
| UBX | ULTRABITHORAX* |
| VND-FL | Full length VND |
| VND-NH | VND containing NK2-specific domain |
| VND/NK | Ventral nervous system defective |

* Protein name is presented in all uppercase letters and non-italicized to help distinguish between the protein and gene symbol^235^.
